# Deciphering the anticoccidial mechanism of Qinghao Changshan formula through network pharmacology and molecular docking

**DOI:** 10.14202/vetworld.2025.2222-2229

**Published:** 2025-08-02

**Authors:** Dong Tian, Hui Fu, Hongxia Tao, Miaolan Li, Qinghua Zhang, Weidong Deng

**Affiliations:** 1 Yunnan Provincial Key Laboratory of Animal Nutrition and Feed, Faculty of Animal Science and Technology, Yunnan Agricultural University, Kunming 650201, China; 2 Hangzhou Xiaoshan Donghai Aquaculture Co., Ltd, Hangzhou, Zhejiang Province 311200, China

**Keywords:** coccidiosis, molecular docking, network pharmacology, poultry parasitology, Qinghao Changshan formula, traditional Chinese medicine

## Abstract

**Background and Aim:**

Coccidiosis is a widespread protozoan disease that severely impacts poultry health and productivity. The Qinghao Changshan (QHCS) formula, composed of multiple traditional Chinese medicinal herbs, is widely used in China for coccidiosis control. Despite its proven clinical efficacy, the molecular mechanisms underlying its therapeutic action remain poorly understood. This study aimed to elucidate the active components and molecular mechanisms of QHCS against coccidiosis using an integrated approach combining network pharmacology and molecular docking.

**Materials and Methods:**

Active compounds of QHCS were identified from public pharmacological databases based on criteria of oral bioavailability ≥ioa and drug-likeness ≥rug-l Targets of these compounds were predicted using SwissTargetPrediction and PharmMapper, and disease-related genes were retrieved from GeneCards, DrugBank, OMIM (Online Mendelian Inheritance in Man), and Therapeutic Target Database. Overlapping targets were visualized using Venn diagrams, and protein–protein interaction (PPI) networks were constructed using STRING and Cytoscape. Gene Ontology (GO) and Kyoto Encyclopedia of Genes and Genomes (KEGG) enrichment analyses were conducted to explore relevant biological functions and pathways. Molecular docking was used to validate interactions between selected active compounds (isorhamnetin, kaempferol, quercetin) and key targets (epidermal growth factor receptor [EGFR], estrogen receptor 1 [ESR1], progesterone receptor [PGR]).

**Results:**

Sixty-nine active compounds and 3476 potential targets of QHCS were identified, with 11 targets overlapping with 87 coccidiosis-related genes. Eight core targets–Amyloid Beta Precursor Protein, interleukin 6, TNF Receptor Associated Factor 1, Platelet Derived Growth Factor Receptor Beta, EGFR, ESR1, Erb-B2 Receptor Tyrosine Kinase 2, and PGR–were identified through PPI network analysis. GO and KEGG enrichment revealed key pathways including focal adhesion, calcium signaling, mitogen-activated protein kinase, ErbB signaling pathway, forkhead box O, and gap junction pathways. Molecular docking confirmed strong binding affinities of isorhamnetin, kaempferol, and quercetin to EGFR, ESR1, and PGR, supporting their regulatory roles in these signaling pathways.

**Conclusion:**

QHCS exhibits anti-coccidial activity by modulating multiple signaling pathways and molecular targets through its key bioactive constituents. These findings provide mechanistic insights into the therapeutic effects of QHCS and lay a theoretical foundation for its broader application in veterinary parasitology.

## INTRODUCTION

Coccidiosis, predominantly caused by *Eimeria* spp., is a protozoan infection that commonly affects the livestock industry, significantly compromising animal health and productivity. The growing intensification of poultry farming has made coccidiosis management increasingly difficult [1]. Current control strategies in poultry farms involve the use of disinfectants, vaccination, medication, and improved management practices [2]. Nevertheless, disinfectants may pose potential hazards to both human and animal health, and incomplete or suboptimal vaccination programs can trigger disease outbreaks. Moreover, the excessive use of antibiotics has contributed to the rise of antimicrobial resistance and the presence of drug residues in animal products [3]. These concerns underscore the urgent need for alternative control methods that are both safe and effective for treating coccidiosis.

Qinghao Changshan (QHCS) is a traditional Chinese herbal formula composed of *Artemisia annua*, *Dichroa febrifuga*, *Astragalus membranaceus*, and *Pulsatilla chinensis*, prepared through double-decoction, filtration, and vacuum concentration techniques [4]. The resulting extract has shown strong anti-coccidial effects and is widely applied in treating coccidiosis across various livestock species, including poultry, ducks, and swine. Previous studies by Zhang *et al*. have confirmed the formulation’s anti-coccidial and hemostatic activities [5]. Compared to standard chemoprophylactic drugs, QHCS offers advantages such as enhanced efficacy, minimal side effects, ease of use, and cost-effectiveness [6]. Despite its promising pharmacological profile, studies on QHCS remain constrained by limited methodologies and insufficient mechanistic insight [7]. Most existing research has focused on individual compounds, often neglecting the potential synergistic effects that may arise from its complex mixture. Therefore, a more detailed exploration of the biochemical mechanisms modulated by QHCS is warranted.

Network pharmacology is an emerging discipline that clarifies the therapeutic mechanisms of traditional Chinese medicine (TCM) formulas by linking multiple active components to multiple disease-related targets through biological networks [8]. This method facilitates the identification of key targets involved in therapeutic processes [9]. In parallel, molecular docking enables the prediction of the binding conformation of small molecules with target proteins, providing insight into potential interactions and binding affinities [10]. Prior studies have effectively utilized the combined approach of network pharmacology and molecular docking to uncover the action mechanisms of various TCM formulas, such as Bu-Shen-Huo-Xue-Fang [11] and Sheng-Mai-San [12].

Although QHCS has demonstrated substantial therapeutic efficacy against coccidiosis in livestock, especially poultry, current knowledge of its pharmacological mechanisms remains fragmented and insufficiently explored. Most prior studies have focused on the isolated effects of individual herbal components without addressing the multi-target, synergistic interactions inherent to TCM formulations. This reductionist approach overlooks the complex biochemical network interactions that likely underlie QHCS’s clinical effectiveness. In addition, despite the proven bioactivity of certain constituents such as artemisinin and halofuginone derivatives, the specific molecular pathways through which QHCS regulates host responses and disrupts the life cycle of *Eimeri*a spp. remain largely undefined. No comprehensive study has yet applied systems biology approaches, such as network pharmacology combined with molecular docking, to systematically decode the bioactive components, their targets, and the key signaling cascades involved in QHCS-mediated anticoccidial action. This methodological gap limits the development of evidence-based, optimized phytotherapeutic stra-tegies and hinders the broader application of QHCS in veterinary parasitology.

To address these limitations, the present study aims to systematically elucidate the active components and underlying molecular mechanisms of the QHCS formula in the treatment of coccidiosis using an integrated approach of network pharmacology and molecular docking. Specifically, the study identifies bioactive compounds of QHCS from pharmacological databases and literature sources, predicts their potential targets, and correlates them with coccidiosis-related genes through protein–protein interaction (PPI) networks. Gene Ontology (GO) and Kyoto Encyclopedia of Genes and Genomes (KEGG) enrichment analyses are employed to determine the biological processes (BPs) and signaling pathways involved. Furthermore, molecular docking is used to validate the binding interactions between core bioactive compounds and target proteins. By combining computational and bioinformatic techniques, this study aims to provide a theoretical framework and mechanistic insights that support the rational application of QHCS as a safe and effective anti-coccidial therapy in livestock.

## MATERIALS AND METHODS

### Ethical approval

The study was approved by the Institutional Animal Care and Use Ethics Committee of Yunnan Agricultural University, Yunnan, China (Approval ID: YAU-LAB-2024-017).

### Study period and location

This study was conducted from February 2024 to September 2024 at the Yunnan Provincial Key Laboratory of Animal Nutrition and Feed, Faculty of Animal Science and Technology, Yunnan Agricultural University, Kunming.

### Identification and prediction of active compounds in TCM formulas

Active constituents from *A. annua, Pulsatilla radix, D. febrifuga*, and *A. membranaceus* were sourced from multiple databases, including TCM integrated database (TCMID, http://www.megabionet.org/tcmid/), Herb database (http://herb.ac.cn/), and TCM systems pharmacology (TCMSP) (http://www.organchem.csdb.cn). Compounds were filtered based on the criteria of oral bioavailability (OB) ≥OB) and drug-likeness (DL) ≥DL)-l In addition, widely studied compounds were incorporated and consolidated for further analysis.

### Construction of the compound-target interaction network

Potential target proteins corresponding to the identified active compounds were predicted using TCMSP, SwissTargetPrediction database (http://swisstargetprediction.ch/), and PharmMapper database (https://www.lilab-ecust.cn/pharmmapper/). The selection criteria included a normalized fit score above 0.9 and the top 15 predicted targets for each compound. Redundant targets were eliminated, and all target names were standardized using the UniProt database (www.uniprot.org). The resulting compound–target interaction network was visualized using Cytoscape version 3.9.0 software (https://cytoscape.org/).

### Identification of coccidiosis-associated targets

Genes related to coccidiosis were collected from GeneCards (https://www.genecards.org/), OMIM (https://www.omim.org/), DrugBank (https://go.drugbank.com/), and the therapeutic target database (TTD) (https://db.idrblab.net/ttd/) using search terms such as “coccidiosis,” “anti-coccidiosis,” and “coccidiosis-infected.” Duplicate entries were removed to obtain a unique list of disease-related genes.

### PPI network analysis

To identify overlapping genes between QHCS-related and coccidiosis-related targets, Venny 2.1.0 (https://www.sthda.com/english/) was used. The shared targets were analyzed in the STRING database (https://cn.string-db.org/) (organism: *Gallus gallus*) with a minimum confidence score of 0.9. The resulting PPI network was visualized using Cytoscape 3.9.0, and key nodes were identified based on CytoNCA (https://apps.cytoscape.org/apps/cytonca) metrics, including degree centrality, betweenness centrality, and closeness centrality.

### Development of the drug–component–disease target network

An integrated network connecting drugs, their active components, and disease-associated targets was constructed using Cytoscape 3.9.0. This visualization enabled the identification of multi-target interactions and polypharmacological properties of QHCS constituents.

### GO and KEGG pathway enrichment analysis

The common targets were subjected to enrich-ment analysis using the DAVID platform (https://davidbioinformatics.nih.gov/) (species: *G. gallus*). GO terms were categorized into BP, molecular function (MF), and cellular component (CC). KEGG pathway enrichment was also performed. Enrichment results with a q < 0.05 were considered statistically significant and visualized through Bioinformatics.com.cn.

### Molecular docking validation

Three key target proteins, epidermal growth factor receptor (EGFR), estrogen receptor 1 (ESR1), and progesterone receptor (PGR), were retrieved from the Protein Data Bank. Ligands were obtained in .mol2 format from PubChem. PyMOL software (https://pymol.org/) was used to remove water molecules and extract native ligands from protein structures. AutoDockTools 1.5.7 (https://autodocksuite.scripps.edu/adt/) was employed to prepare docking parameters using a genetic algorithm (50 runs, 28,000–30,000 generations, and 3 million evaluations). Docking simulations were performed using AutoDock, and binding affinities were calculated. The resulting binding conformations were visualized using PyMOL.

## RESULTS

### Screening of principal active ingredients and prediction of QHCS targets

Chemical constituents of the QHCS formula were retrieved from multiple resources, including TCMID, HERB, Chemistry Database, TCMSP, and relevant published literature. Compounds were selected based on OB ≥Bse and DL ≥Ldedt, a total of 69 bioactive compounds were identified: 21 from *A. annua* L., 11 from *Pulsatillae radix*, 17 from *Dichroae radix*, and 20 from *A. membranaceus* (Fisch.) Bunge (Supplementary Table 1). These were considered as potentially active constituents of QHCS. The TCMSP, SwissTargetPrediction, and PharmMapper databases were then used to predict their target proteins. As a result, 1681 targets were identified for *A. annua* L., 391 for *P. radix*, 175 for *D. radix*, and 1229 for *A. membranaceus*.

### Identification of coccidiosis-related targets and network construction

A comprehensive search of the GeneCards, OMIM, DrugBank, and TTD databases using keywords such as “coccidiosis,” “coccidiosis-infected,” and “against coccidiosis” yielded 87 disease-related genes (Supplementary Table 2).

### Acquisition of overlapping targets

**Figure 1:**
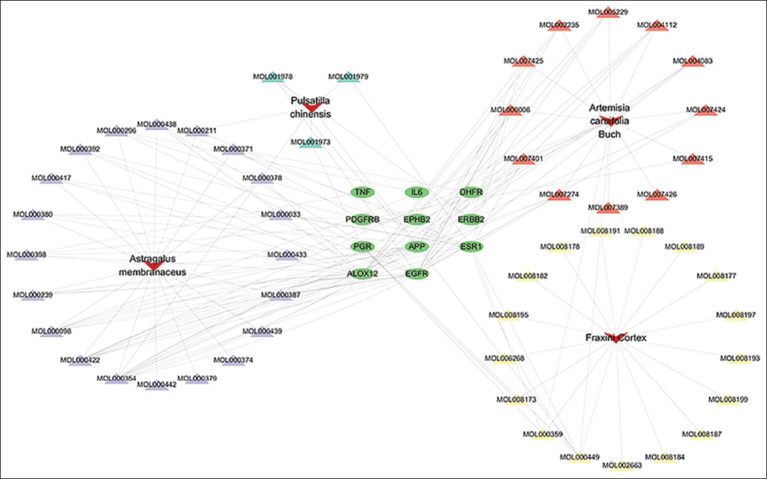
Active ingredient-target-pathway network diagram.

**Figure 2:**
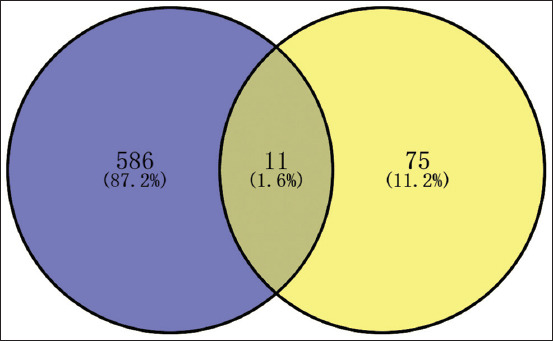
Venn diagram.

**Figure 3:**
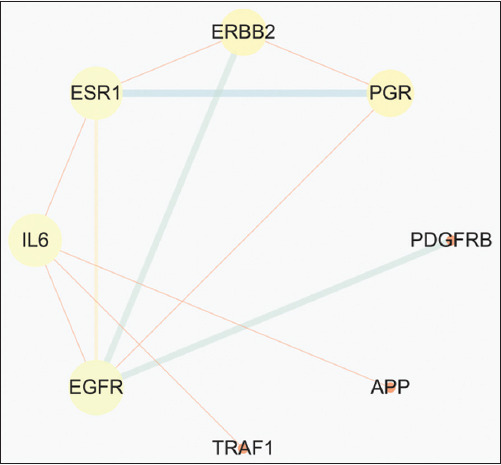
Protein-protein interaction analysis.

A total of 3476 QHCS-related targets and 87 coccidiosis-associated genes were analyzed using Venny 2.1.0 (https://bioinfogp.cnb.csic.es/tools/venny/) to identify overlapping targets (Figures 1 and 2). The analysis revealed 11 shared genes between QHCS and coccidiosis. These overlapping genes were im- ported into the STRING database with a minimum confidence score of 0.9. Subsequent PPI analysis generated a network consisting of 8 core targets: Amyloid Beta Precursor Protein, interleukin 6, TNF Receptor Associated Factor 1, Platelet Derived Growth Factor Receptor Beta, EGFR, ESR1, Erb-B2 Receptor Tyrosine Kinase 2 (ERBB2), and PGR (Figure 3).

### GO enrichment analysis for QHCS in coccidiosis treatment

**Figure 4:**
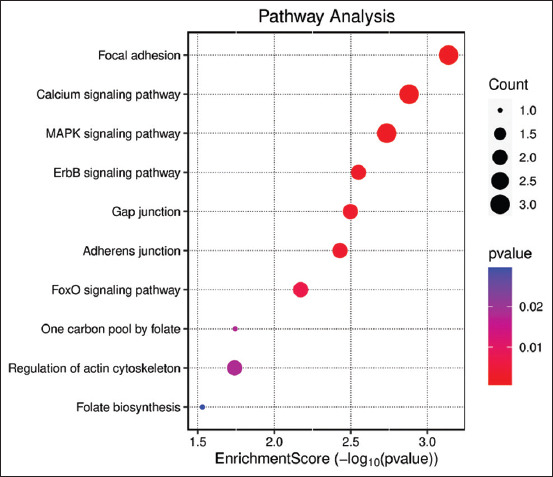
Kyoto encyclopedia of genes and genomes enrichment analysis.

**Figure 5:**
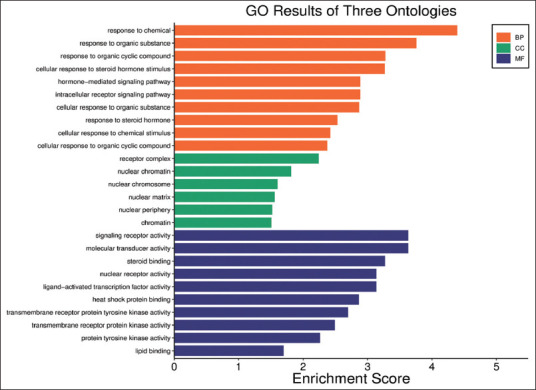
Visualization of gene ontology enrichment.

GO and KEGG enrichment analyses were conduc-ted using the DAVID database for the eight central targets (Figure 4). A total of 3670 GO terms were identified, including 2555 BPs such as “Response to drug,” “Positive regulation of gene expression,” “Transcription,” “DNA-templated,” and “RNA poly-merase II promoter.” The analysis also revealed 45 CCs, including “Integral component of plasma membrane,” “Presynaptic membrane,” and “Membrane raft,” and 60 MFs, such as “Enzyme binding,” “Protein binding,” “Identical protein binding,” and “Neurotransmitter receptor activity.” The most enriched GO terms (with the highest –log10(p) values) are visualized using bar charts in Figure 5, where the x-axis represents the number of enriched targets.

## KEGG pathway enrichment of QHCS in coccidiosis treatment

KEGG pathway enrichment was performed using R software (https://www.r-project.org/) to identify the biological pathways potentially involved in QHCS treatment of coccidiosis. The pathways were sorted by p-value, and a q < 0.05 was set as the cutoff for statistical significance. The top 10 enriched KEGG pathways included Focal Adhesion, Calcium Signaling, mitogen-activated protein kinase (MAPK) signaling, ErbB signaling, gap junction, adherens junction, Forkhead box O (FoxO) signaling, one-carbon pool by folic acid, regulation of actin cytoskeleton, folic acid biosynthesis, and the intestinal immune network.

### Molecular docking validation

**Figure 6:**
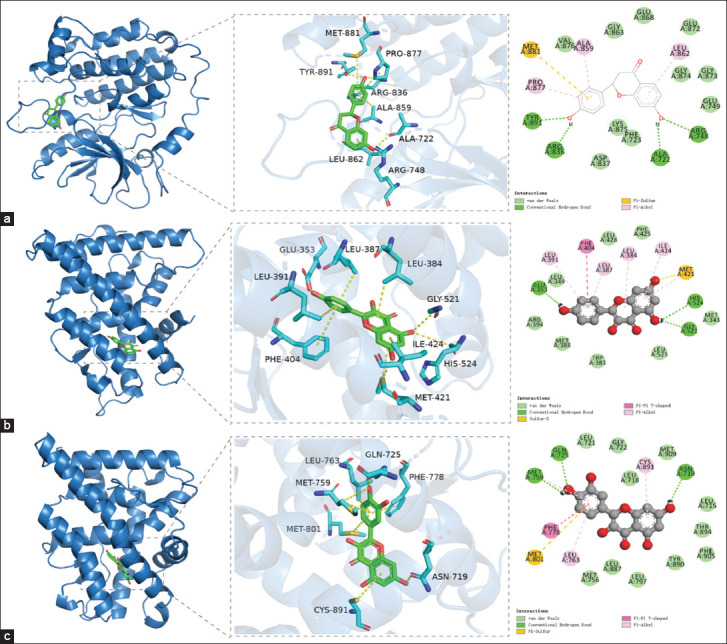
Visualization of molecular docking. (a) Molecular docking of epidermal growth factor receptor, (b) molecular docking of estrogen receptor 1, and (c) molecular docking of progesterone receptor.

To validate interactions between bioactive compounds and disease targets, molecular docking was performed for EGFR, ESR1, and PGR using corresponding compounds and reference drugs. Docking visualizations are shown in Figure 6, and binding scores are presented in Table 1. The docking results showed that ESR1 had strong binding affinities with isorhamnetin and kaempferol, whereas PGR exhibited strong affinity to isorhamnetin and quercetin. In addition, ERBB2 displayed strong binding with isorhamnetin. These findings suggest that QHCS may exert anti-coccidial effects by modulating multiple pathways, including focal adhesion, calcium signaling, MAPK, ErbB, gap junction, adherens junction, and FoxO signaling. The results highlight the potential roles of isorhamnetin, kaempferol, and quercetin in mediating QHCS’s therapeutic action against coccidiosis.

**Table 1:** Molecular docking of isorhamnetin, kaempferol, and quercetin on ESR1, PGR, ERBB2, and EGFR.

Target	Compounds		

	Isorhamnetin (kcal/mol)	Kaempferol (kcal/mol)	Quercetin (kcal/mol)
ESR1	−4.7172	−6.4705	−4.3808
PGR	−5.0622	−4.3985	−6.5936
ERBB2	−5.5217	−4.4286	−4.2464
EGFR	−6.0935	−4.4087	−4.3126

EGFR=Epidermal growth factor receptor, ESR1=Estrogen receptor 1, PGR=Progesterone receptor, ERBB2=Erb-β2 receptor tyrosine kinase 2

## Discussion

### Relevance of QHCS in controlling coccidiosis

Coccidiosis is a widespread parasitic disease affecting various livestock species and remains a significant challenge for modern poultry production systems. QHCS has been widely applied in the prevention and treatment of coccidiosis due to its antiparasitic, growth-promoting, and immunomodulatory effects. Despite its clinical efficacy, the synergistic interactions among the multiple bioactive components of QHCS have not been thoroughly elucidated. In the present study, the chemical constituents of QHCS were compiled through a literature review and public databases. Its potential mechanisms against coccidiosis were explored using network pharmacology, and key active compounds were validated through molecular docking simulations.

### Key bioactive compounds identified by network pharmacology

Network pharmacological analysis revealed that isorhamnetin, kaempferol, and quercetin are the primary active constituents within the QHCS formula. Previous research indicates that isorhamnetin may affect sporulated oocysts by modulating lesion scores and oocyst output. Kaempferol has demonstrated both coccidiocidal and coccidiostatic properties, exhibiting cysticidal activity and inhibiting oocyst sporulation in a dose-dependent fashion, thereby potentially disrupting the parasite’s life cycle [13]. Furthermore, kaempferol has been associated with enhanced weight gain, reduced oocyst shedding, and improved humoral and cellular immune responses. Quercetin, known for its antioxidant properties, may alleviate intestinal lipid peroxidation, suppress oocyst production, lower fecal consistency scores and mortality, and promote weight gain [14]. These reported activities align well with the pharmacological roles attributed to these compounds in the current study.

### Enriched pathways implicated in QHCS therapeutic action

KEGG pathway enrichment of the PPI network suggested that the core targets of QHCS are involved in multiple signaling pathways. These include focal adhesion, calcium signaling, MAPK signaling, ErbB signaling, gap junctions, adherens junctions, FoxO signaling, the one-carbon pool via folic acid, regulation of the actin cytoskeleton, folic acid biosynthesis, and the intestinal immune network for immunoglobulin A production.

### Molecular docking supports target–compound interactions

To verify the interactions between the identified bioactive compounds and critical protein targets, molecular docking analysis was conducted. The results demonstrated that isorhamnetin may influence cellular adhesion and regulate MAPK, ErbB, and FoxO signaling pathways. Kaempferol was shown to interact with targets involved in focal adhesion, calcium signaling, MAPK, ErbB, FoxO, and actin cytoskeleton regulation. Quercetin appeared to exert effects primarily through MAPK, ErbB, and FoxO signaling pathways. These findings corroborate the predicted activities of the compounds based on network pharmacological data and previously published literature.

### Experimental support and need for mechanistic validation

Experimental studies support the pharmacological effects of QHCS. For instance, Pierre *et al*. [15] observed improvements in mental status, feed and water consumption, and fecal consistency in poultry challenged with coccidiosis following QHCS treatment. Similarly, QHCS showed strong anticoccidial efficacy, with anticoccidial indices of 166.6 and 173.8. These dosages significantly boosted chick growth performance, increasing weight gain by 119.8% and 101.6%, respectively [16].

### Future directions and mechanistic considerations

TCM formulations, including QHCS, are based on the principle of synergism among multiple herbs. However, the synergistic interactions integral to this therapeutic philosophy are not fully captured in network pharmacology or molecular docking models. Moving forward, advanced experimental techniques should be applied to isolate and characterize bioactive constituents, elucidate their mechanisms of action, and assess potential toxicological effects. This integrated approach will help establish a robust scientific framework for optimizing and expanding the use of QHCS in veterinary parasitology.

## Conclusion

This study employed an integrative approach combining network pharmacology and molecular docking to elucidate the anti-coccidial mechanisms of the QHCS formula, a traditional Chinese medicinal preparation widely used in poultry disease management. A total of 69 active compounds were identified, with isorhamnetin, kaempferol, and quercetin emerging as the principal bioactive constituents. These compounds were shown to interact with eight key protein targets–including EGFR, ESR1, and PGR–implicated in several signaling cascades such as MAPK, ErbB, Calcium signaling, Focal adhesion, FoxO, and the intestinal immune network. Molecular docking validated strong binding affinities between these compounds and their targets, reinforcing the reliability of the network pharmacology predictions.

Practically, these findings provide a scientific rationale for the continued use of QHCS in veterinary applications, particularly for the treatment and prevention of coccidiosis in poultry. The ability of QHCS to modulate multiple immune and signaling pathways suggests its potential as a multitarget, low-toxicity alternative to synthetic anticoccidial drugs, especially in the context of growing antimicrobial resistance and regulatory restrictions on antibiotic usage.

The strength of this study lies in its systematic and multi-dimensional strategy, which integrates compound screening, target prediction, pathway enrichment, and docking validation–offering a comprehensive understanding of QHCS’s pharmacodynamic profile.

However, several limitations exist. First, the study is based entirely on in silico predictions, lacking experimental validation *in vitro* or *in vivo*. Second, the synergistic interactions between multiple compounds in QHCS–a fundamental principle of TCM–remain underexplored in current computational frameworks.

Looking ahead, future research should focus on experimental validation of the predicted interactions and pathways using cellular and animal models. Moreover, advanced analytical techniques, such as metabolomics and transcriptomics, should be employed to characterize compound synergy, pharmacokinetics, and potential toxicological risks.

In summary, this study offers novel mechanistic insights into how QHCS exerts its anti-coccidial effects, establishing a solid theoretical foundation for its application in veterinary medicine. The findings not only reinforce the therapeutic value of QHCS but also advocate for broader integration of TCM into modern parasitic disease control strategies.

## DATA AVAILABILITY

The supplementary data can be made available from the corresponding author upon request.

## AUTHORS’ CONTRIBUTIONS

DT: Conceptualized and designed the study. HF, HT, and ML: Conducted the experiment, data collection, and revised the manuscript. QZ: Data extraction and statistical analyses. WD: Investigation, validation, and edited the manuscript. All authors have read and approved the final manuscript.
